# Obesity promotes lipid accumulation in lymph node metastasis of gastric cancer: a retrospective case‒control study

**DOI:** 10.1186/s12944-022-01734-7

**Published:** 2022-11-17

**Authors:** Jian Xiao, Kuan Shen, Kanghui Liu, Yuanhang Wang, Hao Fan, Quan Cheng, Xinyi Zhou, Li Hu, Gang Wang, Zekuan Xu, Li Yang

**Affiliations:** 1 Department of General Surgery, The First Affiliated Hospital of Nanjing Medical University, Nanjing, Jiangsu Province China; 2 Department of General Surgery, Liyang People’s Hospital, Liyang Branch Hospital of Jiangsu Province Hospital, Liyang, Jiangsu Province China

**Keywords:** Stomach neoplasms, Lymphatic metastasis, Obesity, Body mass index, Lipids

## Abstract

**Background:**

The connection between obesity, lipid accumulation, and lymph node metastasis (LNM) in gastric cancer (GC) is unclear.

**Methods:**

The association of body mass index (BMI) and serum lipid levels with LNM was measured by calculating the odds ratio (OR) and 95% confidence interval (CI) in 1,058 eligible GC patients with a mean age of 61.4 years. Meanwhile, differentially expressed genes (DEGs) were identified between lymph node metastasis-positive (N +) and -negative (N0) groups using public RNA-seq data. Neutral lipids in human GC samples were detected by Oil red O staining. The expression of cluster of differentiation 36 (CD36), fatty acid synthase (FASN), and lipoprotein lipase (LPL) was detected by immunohistochemistry (IHC) and quantitative real-time PCR.

**Results:**

Compared with normal-weight patients, overweight (OR = 2.02, 95% CI = 1.26–3.23) and obese (OR = 1.83, 95% CI = 1.15–2.91) patients showed increased ORs for LNM. However, no significant results were obtained for serum lipids in the multivariable-adjusted model (*P* > 0.05). Subgroup analysis suggested that increased low-density lipoprotein cholesterol was a risk factor in females (OR = 1.27, 95% CI = 1.02–1.59). Functional enrichment analysis of DEGs revealed a connection between lipid metabolism and LNM. Meanwhile, lipid staining showed a mass of lipids in obese N + tumor samples, and IHC analysis indicated an increase in LPL and CD36 expression in N + cases, implying a crucial role for exogenous lipid supply in LNM.

**Conclusions:**

High BMI significantly increases the risk of LNM in GC and promotes lipid accumulation in GC cells in LNM.

**Supplementary Information:**

The online version contains supplementary material available at 10.1186/s12944-022-01734-7.

## Introduction

Gastric cancer (GC) is one of the deadliest malignancies, with an estimated 769,000 deaths worldwide in 2020 [1]. In recent years, increasing data have suggested that obesity is involved in cancer (such as breast cancer [2], esophageal cancer [3], and GC [4]), diabetes [5], fatty liver [6], and inflammation [7]. Obesity, as a modifiable lifestyle factor, has become a worldwide epidemic, currently affecting > 2 billion people [8]. Hence, much attention has been given to the impact of obesity on cancer incidence, progression, and therapeutic outcomes [9–11]. Of note, complementary therapies based on lipid-lowering agents or anti-obesity pharmacotherapy for cancer have been proposed, given the function of these drugs in reducing inflammation and oxidative stress [12–14]. In GC, the lymph node is the leading metastatic site, and its involvement is deemed an important prognostic factor and can guide crucial clinical decisions of GC patients [15, 16]. However, the association between obesity and LNM remains unclear.

Obesity, as a state of nutrient excess, shows enhanced storage of lipids in adipose tissue and accumulation of serum lipids. Cancer cells experience lipid metabolic changes to fulfill the growing demand for lipids during metastasis [17]. These lipids can be acquired from endogenous synthesis or exogenous sources (for example, adjacent adipocytes and circulating lipids) [18]. Hence, several studies have proposed that obesity can promote tumor progression by reprogramming lipid metabolism in cancer cells [19, 20]. The lymph node is a lipid-rich microenvironment, resulting in a preference for tumor cells to utilize fatty acids as an energy source in metastatic lymph nodes [21]. Meanwhile, cellular and animal models have also shown that primary tumor cells increase fatty acid utilization to promote lymph node metastasis [22]. However, very few current epidemiological studies have investigated the correlation between obesity and LNM in GC, which is important to determine the effect of exogenous lipids on LNM from a clinical perspective.

Fatty acids (FAs) are the main component of lipids, and their acquisition in tumor cells is also achieved in two ways: de novo FA synthesis or exogenous supply [18]. FASN, the enzyme responsible for combining malonyl-CoA and acetyl-CoA to produce the saturated fatty acid palmitate, is the most important lipogenic protein [23]. In addition, CD36 is a key molecule for the exogenous uptake of FAs by serving as the fatty acid receptor [24]. Of note, hydrolyzation of triglyceride in circulating very low-density lipoproteins (VLDLs) or chylomicrons by LPL is critical for the uptake of circulating lipids [25]. Thus, we explored the expression levels of these three proteins in GC tissues to reveal the mediators of obesity-related lipid accumulation. Moreover, using RNA sequencing data from The Cancer Genome Atlas (TCGA) program and the Gene Expression Omnibus (GEO) database, we carried out bioinformatic analysis by classifying GC patients into pathological lymph node metastasis-positive (N +) or -negative (N0) groups. Thus, we determined whether lipid metabolism-related genes or pathways influence LNM and validated the role of lipids in LNM from a gene regulation perspective.

In this study, we investigated the associations of preoperative BMI and serum lipids with LNM in 1,058 GC patients. In addition, the existence of lipid accumulation and lipid metabolism abnormalities in LNM was identified.

## Materials and methods

### Patients for the case‒control study

From December 2016 to June 2021, 1,422 primary GC patients were first enrolled in our study. All patients were treated with total, proximal, or distal D2 gastrectomy. The Ethics Committee of the First Affiliated Hospital of Nanjing Medical University approved this study.

Patients were included if they had a histological diagnosis of gastric adenocarcinoma and complete information about BMI, serum lipid levels, and lymph node status. However, cases with the following conditions were excluded: 1) history of neoadjuvant or conversion therapy for GC before surgery; 2) gastric stump carcinoma; 3) history of other primary tumors; 4) usage of lipid-lowering agents; and 5) history of liver disease. Ultimately, 1,215 eligible GC patients were included. Given the impact of age and sex on obesity, we carried out 1:1 propensity score matching based on age and sex. Finally, this study included 1,058 patients.

### Data collection and measurements

Pathologic data were collected, including depth of tumor invasion (T1, T2, T3, and T4), tumor size (the longest diameter), lymph node status (positive or negative), tumor location (upper, middle, and lower), tumor grade (G1, G1-2, G2, G2-3, and G3), perineural invasion status, lymphovascular invasion status, number of examined lymph node status (ELN), and Lauren classification type (intestinal, mixed, and diffuse type). In addition, demographic data were also collected, such as age and sex.

In this study, preoperative BMI was calculated by dividing weight in kilograms by the square of height in meters. According to the WHO classification in Asia, BMI was grouped as follows: underweight (*n* = 40, < 18.5 kg/m^2^), normal weight (*n* = 423, 18.5–22.9 kg/m^2^), overweight (*n* = 288, 23–24.9 kg/m^2^), and obese (*n* = 307, > 25 kg/m^2^) [26]. Of note, we combined underweight and normal-weight patients into one group due to limited statistical power for the underweight group.

After overnight fasting, preoperative venous blood samples were collected in the early morning. The profiles of serum lipids, including total cholesterol (TC, mmol/L), triglycerides (TGs, mmol/L), high-density lipoprotein cholesterol (HDL-C, mmol/L), low-density lipoprotein cholesterol (LDL-C, mmol/L), and lipoprotein (a) (LP (a), mg/L), were measured by an automated analyzer using standard methods. Moreover, the TG/HDL-C ratio (THR) was also calculated [27]. Lipids were assessed both continuously and as ordinal categorical values determined by quartile.

### Bioinformatic analysis

RNA expression data of 375 GC patients from the TCGA database were downloaded using the R package *TCGAbiolinks*, and relative clinicopathological features were retrieved from the cBioPortal program (*n* = 372) [28]. However, 55 cases were excluded from the analysis for the following reasons: 1) received neoadjuvant therapy (*n* = 0); 2) missing information on nodal status (*n* = 17); and 3) had distant metastasis or unknown metastatic status (*n* = 38). We then identified DEGs between N0 (*n* = 103) and N + (*n* = 214) samples using the package *DEseq2* in R software, with a cutoff value of |log_2_ fold change|> 1 and false discovery rate < 0.05. LNM-related DEGs were also detected in the GSE15459 (*n* = 120 for the N + group, *n* = 40 for the N0 group) and GSE84437 (*n* = 353 for the N + group, *n* = 80 for the N0 group) datasets using the R package *limma*. Volcano plot representation of DEGs was achieved by performing the R package *ggplot2*. Gene Ontology (GO) enrichment analyses were conducted using the R package *clusterProfiler*.

### Tissue samples and Oil red O staining

We collected tumor tissue and adjacent normal tissue from twenty-five GC patients who received radical gastrectomy at the First Affiliated Hospital of Nanjing Medical University. None of the patients received any treatment before surgery. Frozen sections of tissues were immediately subjected to lipid staining.

Oil red O staining was performed according to the manufacturer’s protocol (Cat No: C0158S, Beyotime, Shanghai, China). In brief, tissue sections were incubated with staining wash solution for 20 s and then incubated with working solution at room temperature for 30 min. Then, nuclei were stained with hematoxylin for 3 min. The lipid droplets were visualized using a bright-field microscope (Olympus CKX41, Japan).

### RNA extraction and quantitative RT‒PCR assays

Then, quantitative RT‒PCR assays were performed according to previous procedures [29]. The results were standardized to the expression of  β-actin. The specific primers used in this study were as follows: FASN-forward 5′-CGCGTGGCCGGCTACTCCTAC-3′ and FASN-reverse 5′-CGGCTGCCACACGCTCCTCT-3′, LPL-forward 5′- GCAGGAAGUCUGACCAAUATT-3′ and LPL-reverse 5′- UAUUGGUCAGACUUCCUGCTT-3′, β-actin-forward 5’-GCATCGTCACCAACTGGGAC-3’ and β-actin-reverse 5’-ACCTGG CCGTCAGGCAGCTC-3′, CD36-forward 5’-CTTTGGCTTAATGAGACTGGGAC-3' and CD36-reverse 5'-GCAACAAACATCACCACACCA-3', carnitine palmitoyl transferase 1A (CPT1A)-forward 5′-TTCAGTTCACGGTCACTCCG-3′ and CPT1A-reverse 5′-TGACCACGTTCTTCGTCTGG-3′.

### Immunohistochemistry

Immunohistochemistry was conducted according to a previous protocol [30]. Antibodies against FASN (diluted 1:2000, Cat No:10624–2-AP, Proteintech, Wuhan, China), LPL (diluted 1:1000, Cat No: MB63853, Bioworld Technology, Nanjing, China), and CD36 (diluted 1:2000, Cat No: 18836–1-AP, Proteintech, Wuhan, China) were used. A staining score was calculated with a final score of ≤ 4 defined as negative staining and > 4 defined as positive staining [31].

### Statistical analysis

Comparisons of baseline characteristics were conducted using the Pearson chi-squared test or unpaired Student’s t test. Odds ratios (ORs) and 95% confidence intervals (CIs) were calculated using the logistic regression model. Before variables entered the model, we detected linear relationships between the continuous independent variables and the logit transformation value of the dependent variable using the Box-Tidwell method. Restricted cubic spline (RCS) analysis was applied to explore the nonlinear association between continuous BMI and lymph node status, with knots at equally spaced percentiles. *P* for interaction in subgroup analysis was obtained after the incorporation of two-factor interaction terms. SPSS version 22.0 and R version 4.1.3 were used to conduct all statistical analyses.

## Results

### Study characteristics

The case‒control study included 1,058 GC patients, with 529 cases with LNM and 529 controls without LNM. As summarized in Table 1, no differences were detected in sex, age, tumor location, TG, LDL-C, LP (a), or THR (all *P* > 0.05). However, the N + group presented with higher BMI (*P* < 0.001) and lower levels of TC and HDL-C (*P* < 0.001) than the N0 group. Consistent with other literature [32, 33], the N + group was accompanied by poorer differentiation, larger tumor size, higher percentage of perineural invasion and lymphovascular invasion, and higher T stage (all *P* < 0.001). In addition, the N + group showed a lower percentage of intestinal-type and more harvested lymph nodes (all *P* < 0.001).

**Table 1:** Baseline characteristics of subjects by lymph node metastatic status

Variables	Total (*n* = 1058)	N0 (*n* = 529)	N + (*n* = 529)	*P* value
Sex (*n* (%))				0.788
Male	742 (70.1)	373 (70.5)	369 (69.8)	
Female	316 (29.9)	156 (29.5)	160 (30.2)	
Age (*n* (%))				0.116
≤ 65 years	639 (60.4)	332 (62.8)	307 (58.0)	
> 65 years	419 (39.6)	197 (37.2)	222 (40.2)	
Location (*n* (%))				0.108
Upper	312 (29.5)	169 (31.9)	143 (27.0)	
Middle	375 (35.4)	189 (35.7)	186 (35.2)	
Lower	371 (35.1)	171 (32.4)	200 (37.8)	
Grade (*n* (%)) ^†^				** < 0.001**
G1/G1-2	73 (7.0)	71(13.6)	2 (0.4)	
G2	260 (25.0)	188 (35.9)	72 (14.0)	
G2-3	283 (27.3)	105 (20.1)	178 (34.5)	
G3	423 (40.7)	159 (30.4)	264 (51.1)	
Tumor size (*n* (%))				** < 0.001**
≤ 2.5 cm	481 (46.5)	341 (66.5)	140 (26.8)	
> 2.5 cm	554 (53.5)	172 (33.5)	382 (73.2)	
PNI (*n* (%))				** < 0.001**
Absent	685 (64.7)	453 (85.6)	232 (43.9)	
Present	373 (35.3)	76 (14.4)	297 (56.1)	
LVI (*n* (%))				** < 0.001**
Absent	698 (66.0)	472 (89.2)	226 (42.7)	
Present	360 (34.0)	57 (10.8)	303 (57.3)	
T stage (*n* (%))				** < 0.001**
T1	430 (40.6)	354 (66.9)	76 (14.4)	
T2	157 (14.9)	81 (15.3)	76 (14.4)	
T3	325 (30.7)	76 (14.4)	249 (47.1)	
T4	146 (13.8)	18 (3.4)	128 (24.2)	
Lauren (*n* (%)) ^‡^				** < 0.001**
Intestinal	401 (43.8)	257 (58.0)	144 (30.4)	
Diffuse	210 (22.9)	89 (20.1)	121 (25.6)	
Mixed	305 (33.3)	97 (21.9)	208 (44.0)	
ELN	43.21 (11.85)	41.98 (11.59)	44.43 (12.00)	** < 0.001**
TC (mmol/L)	4.49 (0.98)	4.58 (0.97)	4.38 (0.99)	**0.001**
TG (mmol/L)	1.42 (1.07)	1.45 (1.27)	1.38 (0.82)	0.362
HDL-C (mmol/L)	1.11 (0.27)	1.15 (0.28)	1.05 (0.24)	** < 0.001**
LDL-C (mmol/L)	2.78 (0.72)	2.83 (0.71)	2.74 (0.73)	0.060
LP (a) (mg/L)	240.9 (230.6)	232.7 (226.8)	249.2 (234.2)	0.244
THR	1.41 (1.37)	1.41 (1.65)	1.42 (1.02)	0.842
BMI (kg/m^2^)	23.54 (2.99)	23.22 (2.90)	23.84 (3.06)	**0.001**
BMI (*n* (%))				** < 0.001**
< 23 kg/m^2^	463 (43.8)	263 (49.7)	200 (37.8)	
23–24.9 kg/m^2^	288 (27.2)	135 (25.5)	153 (28.9)	
≥ 25 kg/m^2^	307 (29.0)	131 (24.8)	176 (33.3)	

Data are presented as the mean ± standard deviation for continuous variables and number (percentage) for categorical variables. *P* values were calculated using the chi-square test or Student's t test with two -tailed tests. The significant results (*P* < 0.05) are in bold

Abbreviations: *PNI* perineural invasion, *LVI* lymphovascular invasion, *ELN* examined lymph nodes, *TC* total cholesterol, *TG* triglyceride, *HDL-C* high-density lipoprotein cholesterol, *LDL-C* low-density lipoprotein cholesterol, *LP (a)* lipoprotein (a), *THR* triglyceride to high-density lipoprotein cholesterol (TG/HDL-C) ratio, *BMI* body mass index

^†^Missing values for 19 patients

^‡^the information was not recorded in 142 patients

### Odds ratio for LNM risk by BMI and serum lipids

Table 2 shows the associations of categorized BMI and serum lipids with LNM. First, in the univariate logistic analysis, the ORs for LNM increased across the BMI groups (overweight vs. < 23 kg/m^2^, OR = 1.49, *P* = 0.008; obese vs. < 23 kg/m^2^, OR = 1.77, *P* < 0.001). Meanwhile, the OR for LNM in the comparison between the highest and lowest quartiles of TC was also significant (OR = 0.63, *P* = 0.009). Compared with the first quartile, the fourth LP (a) (OR = 1.42, *P* = 0.046) and THR (OR = 1.72, *P* = 0.002) quartiles showed an increased risk of LNM. In contrast, the third and fourth quartiles of HDL-C showed a protective effect on LNM (OR = 0.63, *P* = 0.007 for Q3; OR = 0.39, *P* < 0.001 for Q4). However, in the multivariate-adjusted model, only BMI groups showed significant results: patients in the overweight (OR = 2.02, 95% CI = 1.26–3.23, *P* = 0.003) and obesity (OR = 1.83, 95% CI = 1.15–2.91, *P* = 0.011) groups were associated with a higher risk of LNM (*P* for trend = 0.006). Hence, these results indicated that BMI was an independent risk factor for LNM in GC.

**Table 2:** Association of BMI (categorized) and serum lipid level (by quartiles) with lymph node metastasis

Variables	Univariate logistic analysis		Multivariate logistic analysis ^†^		
	OR (95% CI)	*P* value	OR (95% CI)	*P* value	*P* for trend
TC (mmol/L)					0.949
Q1 (≤ 3.82)	1.00 (ref)		1.00 (ref)		
Q2 (3.83–4.43)	0.77 (0.55–1.08)	0.131	1.48 (0.69–3.18)	0.310	
Q3 (4.44–5.07)	0.88(0.63–1.24)	0.463	1.17 (0.46–2.99)	0.741	
Q4 (≥ 5.08)	0.63 (0.45–0.89)	**0.009**	0.88 (0.26–2.91)	0.831	
TG (mmol/L)					
Q1 (≤ 0.87)	1.00 (ref)				
Q2 (0.88–1.18)	1.05 (0.75–1.47)	0.794			
Q3 (1.19–1.65)	1.11 (0.79–1.57)	0.542			
Q4 (≥ 1.66)	1.09 (0.78–1.54)	0.603			
HDL-C (mmol/L)					0.174
Q1 (≤ 0.92)	1.00 (ref)		1.00 (ref)		
Q2 (0.93–1.08)	0.71 (0.51–1.00)	0.050	0.78 (0.45–1.36)	0.381	
Q3 (1.09–1.27)	0.62 (0.44–0.88)	**0.007**	0.75 (0.40–1.43)	0.385	
Q4 (≥ 1.28)	0.39 (0.27–0.56)	** < 0.001**	0.60 (0.27–1.30)	0.196	
LDL-C (mmol/L)					0.905
Q1 (≤ 2.28)	1.00 (ref)		1.00 (ref)		
Q2 (2.29–2.76)	0.79 (0.56–1.11)	0.182	0.71 (0.34–1.47)	0.351	
Q3 (2.77–3.22)	0.90 (0.64–1.26)	0.524	1.08 (0.44–2.69)	0.864	
Q4 (≥ 3.23)	0.73 (0.52–1.02)	0.069	1.11 (0.36–3.41)	0.858	
LP (a) (mg/L)					0.356
Q1 (≤ 84)	1.00 (ref)		1.00 (ref)		
Q2 (85–161)	1.33 (0.95–1.87)	0.100	1.08 (0.71–1.97)	0.513	
Q3 (162–314)	1.20 (0.85–1.69)	0.303	0.71 (0.42–1.18)	0.187	
Q4 (≥ 315)	1.42 (1.01–1.99)	**0.046**	1.34 (0.79–2.27)	0.277	
THR					0.977
Q1 (≤ 0.74)	1.00 (ref)		1.00 (ref)		
Q2 (0.75–1.12)	1.34 (0.95–1.89)	0.091	1.03 (0.60–1.75)	0.921	
Q3 (1.13–1.66)	1.33 (0.95–1.88)	0.101	0.74 (0.40–1.37)	0.338	
Q4 (≥ 1.67)	1.72 (1.22–2.42)	**0.002**	0.78 (0.39–1.56)	0.482	
BMI (kg/m^2^)					**0.006**
< 23	1.00 (ref)		1.00 (ref)		
23–24.9	1.49 (1.11–2.00)	**0.008**	2.02 (1.26–3.23)	**0.003**	
≥ 25	1.77 (1.32–2.36)	** < 0.001**	1.83 (1.15–2.91)	**0.011**	
Sex					
Male	1.00 (ref)				
Female	1.04 (0.80–1.35)	0.788			
Age (years)					
≤ 65	1.00 (ref)				
> 65	1.22 (0.95–1.56)	0.116			
Location					
Upper	1.00 (ref)		1.00 (ref)		
Middle	1.16 (0.86–1.57)	0.325	1.48 (0.92–2.37)	0.106	
Lower	1.38 (1.02–1.87)	**0.036**	2.06 (1.28–3.31)	**0.003**	
Grade ^‡^					
G1/G1-2	1.00 (ref)		1.00 (ref)		
G2	13.60 (3.25–56.89)	** < 0.001**	3.96 (0.51–30.65)	0.187	
G2-3	60.18 (14.46–250.44)	** < 0.001**	5.44 (0.69–42.95)	0.108	
G3	58.94 (14.26–243.62)	** < 0.001**	5.35 (0.65–43.82)	0.118	
Tumor size (cm)					
≤ 2.5	1.00 (ref)		1.00 (ref)		
> 2.5	5.41 (4.14–7.06)	** < 0.001**	1.47 (0.97–2.20)	0.066	
T stage					
T1	1.00 (ref)		1.00 (ref)		
T2	4.37 (2.93–6.51)	** < 0.001**	1.94 (1.16–3.250	**0.012**	
T3	15.26 (10.68–21.81)	** < 0.001**	4.88 (2.85–8.34)	** < 0.001**	
T4	33.12 (19.07–57.53)	** < 0.001**	8.43 (4.06–17.48)	** < 0.001**	
PNI					
Absent	1.00 (ref)		1.00 (ref)		
Present	7.63 (5.67–10.27)	** < 0.001**	2.18 (1.39–3.43)	**0.001**	
LVI					
Absent	1.00 (ref)		1.00 (ref)		
Present	11.10 (8.03–15.36)	** < 0.001**	4.45 (2.92–6.79)	** < 0.001**	
Lauren ^§^					
Intestinal	1.00 (ref)		1.00 (ref)		
Diffuse	2.43 (1.72–3.41)	** < 0.001**	1.22 (0.60–2.48)	0.585	
Mixed	3.83 (2.79–5.25)	** < 0.001**	2.59 (1.51–4.42)	**0.001**	
ELN	1.02 (1.01–1.03)	**0.001**	1.01 (1.00–1.03)	0.119	

Abbreviations: *OR* Odds ratio, *CI* confidence interval, *Q* quartile, *TC* total cholesterol, *TG* triglyceride, *HDL-C* high-density lipoprotein cholesterol, *LDL-C* low-density lipoprotein cholesterol, *LP (a)* lipoprotein (a), *THR* TG/HDL-C, *BMI* body mass index, *PNI* perineural invasion, *LVI* lymphovascular invasion, *ELN* examined lymph nodes

^†^ Adjusted for TC, HDL-C, LDL-C, LP (a), THR, BMI, location, grade, T stage, tumor size, PNI, LVI, Lauren classification, and ELN

^‡^ Missing values for 19 patients

^§^ Missing values for 142 patients

The significant results are in bold

When BMI and serum lipids were included in the univariate logistic regression model as continuous variables, TC (OR for 0.1 mmol/L increase = 0.98, 95% CI = 0.97–0.99) and HDL-C (OR for 0.1 mmol/L increase = 0.87, 95% CI = 0.82–0.91) still showed a negative correlation with LNM while a high BMI indicated an increased risk of LNM (OR for 5 kg/m^2^ increase = 1.43, 95% CI = 1.16–1.75) (Table 3). Of note, BMI, TC, and HDL-C did not obtain significant results after adjustment for covariates (*P* > 0.05). However, a marginal effect of BMI on LNM could be detected (OR for 5 kg/m^2^ increase = 1.39, 95% CI = 1.00–1.93, *P* = 0.050) (Table 3).

**Table 3:** Odds ratio for lymph node metastasis by increased BMI (continuous) and serum lipid level (continuous)

Continuous Variables	Univariate logistic analysis		Multivariate logistic analysis ^†^	
	OR (95% CI)	*P* value	OR (95% CI)	*P* value
TC (mmol/L) ^a^	0.98 (0.97–0.99)	**0.001**	0.99 (0.97–1.01)	0.545
TG (mmol/L) ^a^	0.99 (0.98–1.01)	0.366		
HDL-C (mmol/L) ^a^	0.87 (0.82–0.91)	** < 0.001**	0.92 (0.85–1.01)	0.089
LDL-C (mmol/L) ^a^	0.98 (0.97–1.00)	0.060		
LP (a) (mg/L) ^b^	1.00 (0.99–1.01)	0.244		
THR ^c^	1.01 (0.90–1.14)	0.842		
BMI (kg/m^2^) ^d^	1.43 (1.16–1.75)	**0.001**	1.39 (1.00–1.93)	0.050

^†^ Adjusted for TC, HDL-C, BMI, age, sex, location, grade, T stage, PNI, LVI, Lauren classification, tumor size, and ELN

^a^ Odds ratios reported for a 0.1 mmol/L increase in TC, TG, HDL-C, and LDL-C

^b^ Odds ratios reported for a 10 mg/L increase in LP (a)

^c^ Odds ratios reported per standard deviation (SD) increment

^d^ Odds ratios reported for a 5 kg/m^2^ increase

The significant results are in bold

Using the RCS model with 23 kg/m^2^ as the reference, we failed to find a nonlinear relationship between BMI and LNM risk irrespective of variables included in the model for adjustment (*P* for nonlinear > 0.05; Figure S1).

### Subgroup analysis based on BMI and serum lipids

As continuous variables, LP (a), LDL-C, and BMI were associated with LNM in certain groups (Table 4). Higher LP (a) showed a slightly increased risk of LNM in older persons (OR for 10 mg/L increase = 1.02, 95% CI = 1.01–1.03, *P* = 0.022) and in lower stomach tumors (OR for 10 mg/L increase = 1.02, 95% CI = 1.01–1.03, *P* = 0.031), while the association between LDL-C and LNM was only evident in females (OR for 0.1 mmol/L increase = 1.27, 95% CI = 1.02–1.59, *P* = 0.037). Meanwhile, BMI was an independent risk factor in males (OR for 5 kg/m^2^ increase = 1.69, 95% CI = 1.12–2.55, *P* = 0.013), lower stomach tumors (OR for 5 kg/m^2^ increase = 1.69, 95% CI = 1.03–2.77, *P* = 0.037), and tumors with moderately to poorly differentiated grade (OR for 5 kg/m^2^ increase = 2.43, 95% CI = 1.32–4.46, *P* = 0.004).

**Table 4:** Associations of continuous variables (LP (a), BMI, and LDL-C) with lymph node metastasis in subgroup analysis

Variables	LP (a) ^a^		BMI ^b^		LDL-C ^c^	
	OR (95% CI) ^†^	*P *^†^	OR (95% CI) ^†^	*P *^†^	OR (95% CI) ^†^	*P *^†^
Age (years)						
≤ 65	0.99 (0.98–1.00)	0.256	1.47 (0.94–2.30)	0.092	0.99 (0.98–1.00)	0.256
> 65	1.02 (1.01–1.03)	**0.022**	1.57 (0.90–2.73)	0.109	1.10 (0.92–1.32)	0.304
Sex						
Male	1.00 (0.99–1.01)	0.296	1.69 (1.12–2.55)	**0.013**	1.04 (0.90–1.19)	0.602
Female	1.00 (0.98–1.02)	0.992	1.16 (0.62–2.18)	0.640	1.27 (1.02–1.59)	**0.037**
Location						
Upper	0.99 (0.97–1.01)	0.390	1.21 (0.52–2.78)	0.662	1.25 (0.96–1.64)	0.094
Middle	0.99 (0.98–1.01)	0.608	1.51 (0.82–2.79)	0.189	1.10 (0.91–1.33)	0.340
Lower	1.02 (1.01–1.03)	**0.031**	1.69 (1.03–2.77)	**0.037**	1.08 (0.89–1.32)	0.430
T stage						
T1 + T2	1.01 (1.00–1.02)	0.058	1.46 (0.92–2.32)	0.105	1.14 (0.96–1.35)	0.132
T3 + T4	1.00 (0.98–1.01)	0.474	1.44 (0.87–2.38)	0.160	1.13 (0.95–1.34)	0.171
Grade						
G2	1.01 (0.99–1.03)	0.249	0.85 (0.39–1.89)	0.696	1.13 (0.88–1.44)	0.339
G2-3	1.01 (0.99–1.02)	0.279	2.43 (1.32–4.46)	**0.004**	1.04 (0.84–1.30)	0.692
G3	0.99 (0.98–1.01)	0.399	1.29 (0.75–2.22)	0.366	1.21 (0.99–1.48)	0.055

*OR* odds ratio, *CI* confidence interval

^†^ Adjusted for TG, TC, HDL-C, LDL-C, LP (a), THR, BMI, age, sex, location, grade, T stage, tumor size, PNI, LVI, Lauren classification, and ELN (excluding the stratified factor in each stratum)

^a^ Odds ratios reported for a 10 mg/L increase in LP (a)

^b^ Odds ratios reported for a 5 kg/m^2^ increase

^c^ Odds ratios reported for a 0.1 mmol/L increase in LDL-C

The significant results are in bold

The association between BMI groups and LNM risk also differed by age, sex, tumor location, grade, and T stage (Supplementary Table 1). In detail, both the overweight and obesity groups had a greater risk of LNM in males and tumors with moderately to poorly differentiated grades (*P* < 0.05). Furthermore, the overweight group showed a higher risk of LNM in patients older than 65 years (OR = 2.68, *P* = 0.013) or tumors located in the upper stomach (OR = 4.06, *P* = 0.035). Meanwhile, we detected positive correlations between the obesity group and LNM risk in tumors with T1 or T2 stage (OR = 2.05, *P* = 0.031) and tumors in the lower stomach (OR = 2.25, *P* = 0.041). None of the *P* values for the interaction were statistically significant.

### Obesity promotes lipid accumulation in LNM

**Fig. 1:**
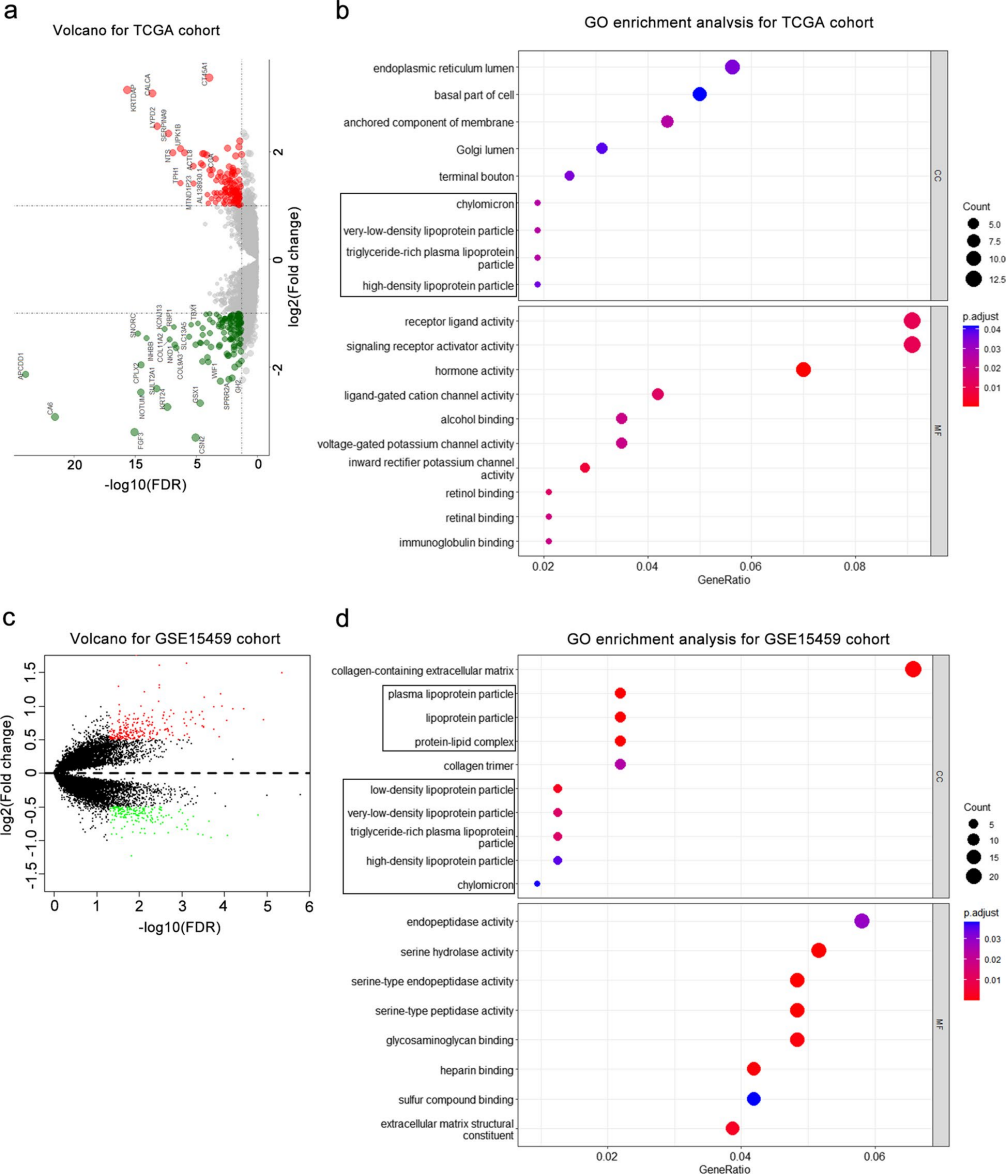
Identification and functional enrichment analysis of DEGs. **a** Volcano plot presentation of DEGs between N0 and N + patients from the TCGA cohort. **b** GO enrichment analysis of DEGs from the TCGA cohort. The part enclosed by the black box is the cellular components related to lipid metabolism. **c** Volcano plot showing DEGs from the GSE15459 cohort. **d** Dot plot showing the results of GO enrichment analysis of DEGs from the GSE15459 cohort

We first sought to investigate whether GC cells undergo metabolic reprogramming to accommodate the high demands of lipids. Using RNA-seq data from the TCGA database, we identified a total of 263 DEGs between the N + and N0 groups (Fig. 1a). Then, these DEGs were subjected to GO enrichment analyses. Notably, ten DEGs showed enrichment in lipid metabolism-related cellular components, such as chylomicrons, VLDL particles, triglyceride-rich plasma lipoprotein particles, and high-density lipoprotein particles (*P* < 0.05; Fig. 1b). Similarly, analysis of GSE15459 also revealed 438 DEGs (|log_2_ FC|> 0.5, *P* < 0.05), and GO analysis of these DEGs showed the same CC enrichments as the TCGA cohort (Fig. 1c, d). In the same manner, GO analysis of DEGs from the GSE84437 cohort exhibited the enrichment of DEGs in molecular functions, such as sterol binding and cholesterol binding (*P* < 0.05; Figure. S2a, b). Thus, these findings indicated a potential role of lipid metabolism abnormalities in LNM of GC.

**Fig. 2:**
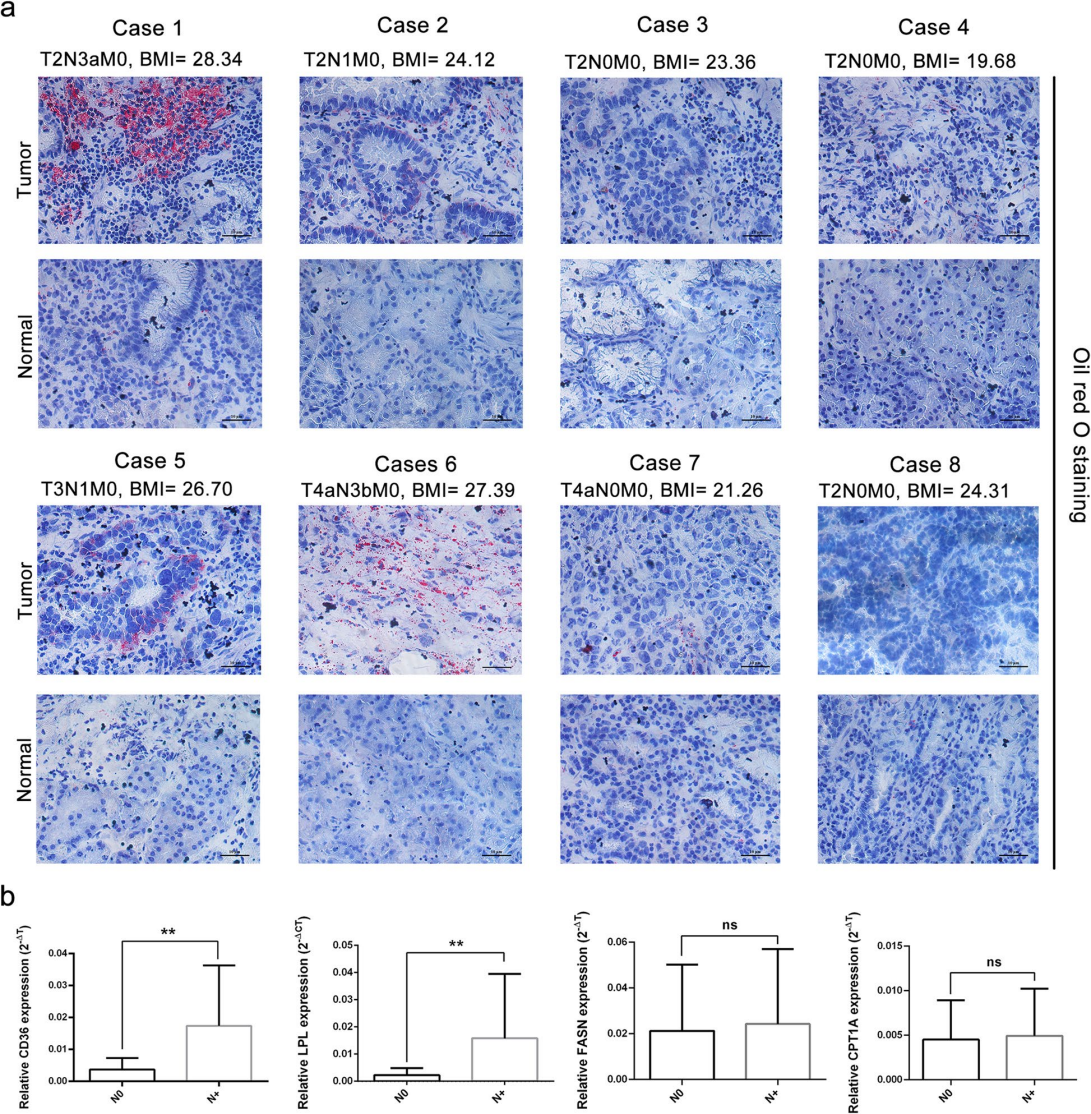
Oil red O staining of lipids in human tissue samples. **a** Detection of lipids in human tissue samples by oil red O staining. Scale bar = 10 µm. Neutral lipids in tissues were dyed red. **b** Expression levels of CD36, LPL, FASN, and CPT1A in forty tumor tissues (N0 = 15, N +  = 25) by qRT‒PCR

Oil red O staining of human tissue samples revealed a mass of lipid droplets in N + tumor samples compared with N0 tumor samples and normal tissue samples, suggesting that GC cells routinely store high levels of lipids for metastasis (Fig. 2a). Meanwhile, we found that a high BMI was correlated with increased lipids in GC tissues (Fig. 2a).

**Fig. 3:**
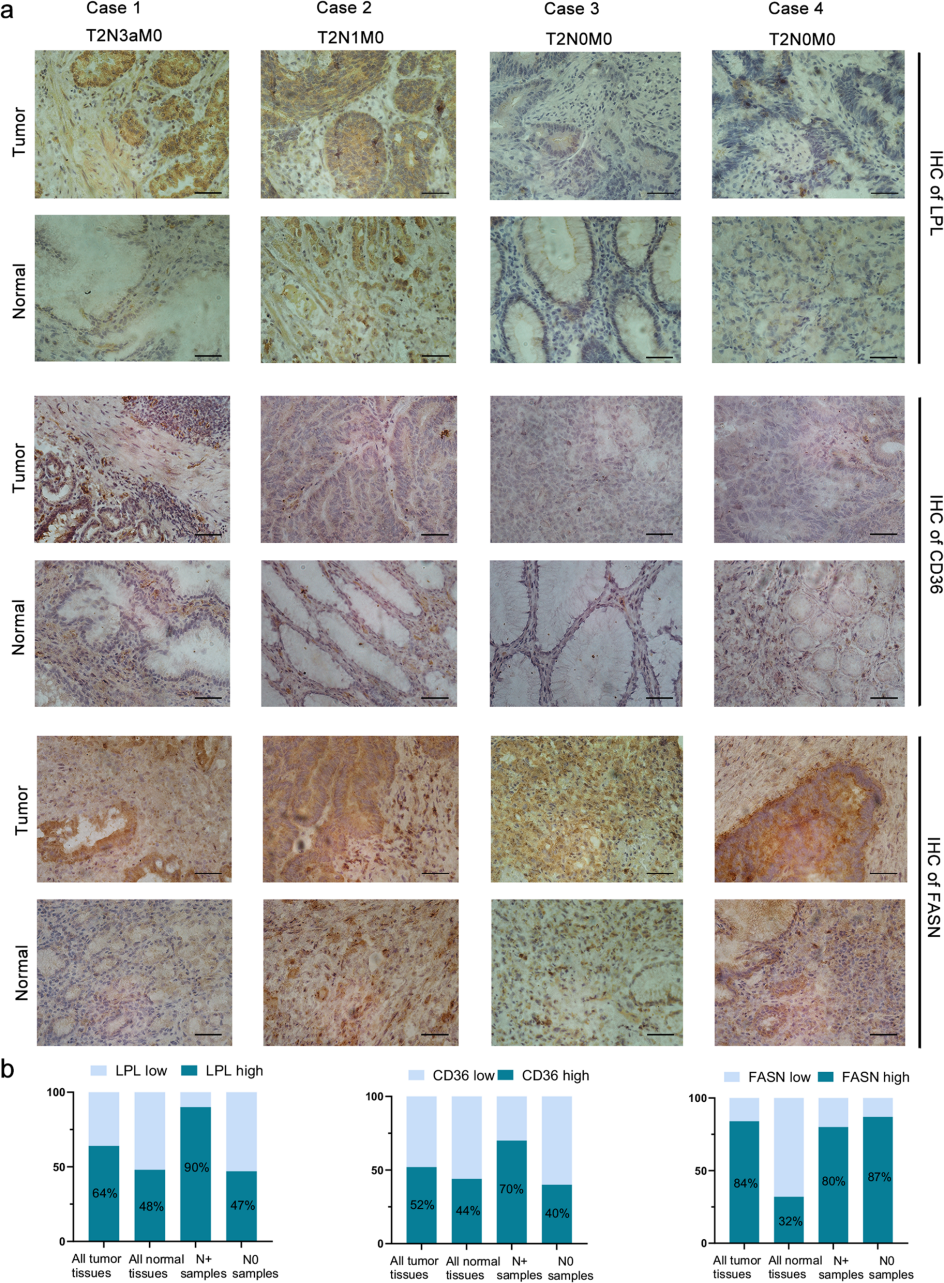
Expression levels of LPL, CD36, and FASN in GC samples. **a** Representative microscopic image of LPL, CD36, and FASN expression in tumor tissues, adjacent normal tissues, N + tumor samples, and N0 tumor samples. Scale bar = 10 µm. **b** Staining intensity of LPL, CD36, and FASN expression was calculated in all tumor samples (*n* = 25), normal tissues (*n* = 25), N + tumor samples (*n* = 10), and N0 samples (*n* = 15)

Lipid metabolism includes lipid uptake, biosynthesis, and lipolysis (such as fatty acid oxidation) [34]. Therefore, to verify the involvement of lipid metabolism in LNM, we detected the expression of CD36 and LPL, FASN, and CPT1A (one of the CPT1 members that serves as the rate-limiting enzyme for long-chain FA entrance and subsequent oxidation) by qRT‒PCR. It showed higher expression levels of CD36 and LPL in N + tumor tissues than in N0 tissues, while no differences were detected for the expression of FASN and CPT1A (Fig. 2b). Meanwhile, we measured the expression of FASN, CD36, and LPL in GC tissues and adjacent normal tissues by IHC to determine which mechanisms of lipid uptake are more evident in LNM (Fig. 3a). Consistent with other literature [23], the expression of FASN was higher in tumor tissues than in normal tissues. However, LPL and CD36 did not show a difference between tumor tissues and adjacent normal tissues (Fig. 3b). Consistent with the qRT-PCR results, higher expression levels of LPL and CD36 were detected in N + samples, while no difference was observed for FASN (Fig. 3b). Thus, these results revealed that LPL/CD36-mediated exogenous FA uptake is one of the primary mechanisms of lipid uptake in LNM in GC cells. These data further strengthened the crucial role of obesity-promoted lipid accumulation in LNM.

## Discussion

In our case‒control study, BMI stratified by the WHO classification was an independent risk factor for LNM of GC. However, serum lipids did not show significant results in any of the cases during the multivariate analyses. GO analyses of three public sequencing databases showed concordant enrichment of DEGs in lipid metabolism-related modules. In addition, lipid accumulation and significant upregulation of LPL and CD36 expression were discerned in N + tumor tissue samples and obesity cases, further strengthening the association of lipid metabolism with LNM. Therefore, we believe that obesity, at least from BMI, is a risk factor for LNM and provides a lipogenic environment in GC.

A positive association between high BMI and LNM risk has been detected in several cancer sites, such as the thyroid, breast, bladder, and prostate [35–38]. However, few studies have explored the relationship between BMI and LNM in GC. In undifferentiated early gastric cancer, Zou et al*.* showed that BMI was a protective factor against LNM [39]. Park et al*.* found that decreased LNM was associated with high visceral obesity but not BMI [40]. The above conclusions were not consistent with our results. One possible explanation is reverse causality: underweight or normal-weight patients may have more advanced disease and greater disease-related weight loss, thus resulting in the illusion of a negative correlation or irrelevance of BMI with LNM [41]. Regrettably, Park et al*.* did not show information about the underweight group and prediagnostic weight loss [40]. Another reason is the limited accessibility to lymph node dissection in obese patients [42]. Park et al*.* reported a mean harvested number of 38.4, and Zou et al*.* did not provide this value [39, 40]. In contrast, our study showed a higher number of dissected lymph nodes, with a mean of 43.2. A third explanation is the relatively small sample size for the two previous studies (*n* = 495 for the Park study, *n* = 323 for the Zou study), resulting in limited statistical power. Finally, various studies have proposed a link in tumor biology between obesity and cancer metastasis [43]. The possible mechanisms of obesity-driven metastasis include increased local and circulating proinflammatory cytokines, upregulated levels of adiponectin and leptin, reprogramming of cellular energetics, insulin resistance, and immune dysfunction [44]. Moreover, several epidemiological studies have indicated positive correlations of high BMI and visceral obesity with peritoneal dissemination of GC [45, 46]. In addition, Li et al*.* found that obese omental adipocytes increase DGAT2 expression and thus promote lipid droplet accumulation and redox balance in the peritoneal metastasis of GC [19]. Overall, we believe obesity may create a microenvironment that promotes LNM in GC, but a population-based, randomized study is needed to prove these findings.

In clinical practice, BMI is frequently employed to evaluate the degree of obesity [26]. Intriguingly, a recent large cohort study revealed an improved prognosis of overweight or moderately obese cases compared with normal-weight patients, indicating the presence of the "obesity paradox" in GC [47]. The typical "obesity paradox" refers to the disconnection between the usual adverse health effects associated with obesity and the significant survival advantage of high BMI in cancer [48, 49]. Hence, researchers encourage further examination of specific body composition metrics (such as muscle tissue mass, visceral fat mass, and subcutaneous fat mass) [41]. Subcutaneous adipose tissue is most often inversely associated with mortality, whereas high visceral fat levels show higher inflammation and poor outcomes [41]. Thus, measures of body composition should be integrated to reveal the multiple associations between preoperative obesity and LNM. Moreover, regarding the relationship between food trends and obesity and the role of diet in cancer patients, an analysis of the impact of dietary factors on the LNM of GC is necessary [50, 51].

Enhanced lipid synthesis, storage, and catabolism are features of tumorigenesis and disease progression [34]. Obesity is known to increase systemic fatty acid availability to cancer cells by increasing lipoprotein-​containing triacylglycerol in circulation or by strengthening the interaction between local adipocytes and cancer cells [10]. Of note, Kitayama et al*.* found that hypertriglyceridemia was an independent risk factor for nodal metastasis in men with early-stage gastric cancer, and Shen et al*.* demonstrated that a low level of HDL-C indicates a high risk of LNM in GC [52, 53]. In our study, an increase in LPL and CD36 expression was detected in obese N + samples, further reflecting the importance of the exogenous supply of fatty acids in LNM. However, the detailed mechanism of how LPL/CD36 is involved in the LNM of GC needs further investigation.

## Study strengths and limitations

The greatest strength of our study was the comprehensive analysis of the relationship between obesity, lipid accumulation, and LNM in terms of epidemiology, histology, and molecular expression. Our study, based on the largest enrolled population as we know, first unraveled an accelerative role of high BMI on nodal metastasis of GC. On the other hand, our study explored a positive association between lipid accumulation and nodal metastasis, which histologically confirmed the role of high BMI in the lipid accumulation of nodal metastasis. In a way, we could think this study found a new characteristic of GC patients with LNM, lipid accumulation in primary cancer tissues. In addition, the high expression level of CD36 and LPL in N + samples further verified the importance of an exogenous supply of lipids in LNM, reflecting the impact of obesity on nodal metastasis in another way. However, several limitations in this study should not be neglected. First, this study was conducted based on retrospective information from one medical center, which might introduce selection biases and influence the results. Second, other obesity-related factors, such as body composition metrics, dietary habits, and prediagnostic weight loss, were not included in this study. Third, the sample size of the underweight group was relatively small, limiting our further analysis of the relationship between obesity and LNM [54]. Fourth, contrary to the results of others, we did not find a correlation between serum lipids and LNM, highlighting the need for a population-based, randomized study. Finally, the detailed mechanism of how obesity promotes lipid accumulation in LNM and the role of LPL/CD36 in this process were not investigated.

## Conclusions

This study revealed a positive correlation between preoperative BMI and LNM in GC. This conclusion emphasizes the value of BMI in predicting nodal metastasis and suggests that normal weight might be of benefit in preventing metastasis in certain cancers. In addition, the bioinformatic analysis and lipid staining in tissue samples further strengthened the involvement of lipid metabolism in the biology of LNM.

## Supplementary Information

**Additional file 1: Figure S1.** Detection of nonlinear relationships between BMI and lymph node metastasis. **a** Using the restricted cubic spine (RCS) function in the logistic regression model did not observe a nonlinear association between the BMI and lymph node metastasis risk. **b** RCS analysis with adjustment of sex, age, tumor location, grade, T stage, PNI, LVI, ELN, tumor size, and Lauren type. **FigureS2.** Bioinformatic analysis of the GSE84437 cohort. **a** Identification of DEGs between N0 and N+ patients in the GSE84437 cohort. **b** GO enrichment analysis of DEGs that were derived from the GSE84437 cohort.

**Additional file 2: Supplementary Table 1.** Subgroup analysis for the association between categorized BMI and lymph node metastasis.

## Data Availability

The RNA-seq data can be downloaded at https://portal.gdc.cancer.gov/, https://www.ncbi.nlm.nih.gov/geo/ (GSE84437, GSE15459). The code used and the case‒control data used during the current study are available from the corresponding author upon reasonable request.
