# Supplementary material for: Obesity promotes lipid accumulation in lymph node metastasis of gastric cancer: a retrospective case‒control study
Source: Lipids Health Dis. 2022 Nov 17;21:123. doi: 10.1186/s12944-022-01734-7 (PMC9673345; doi:10.1186/s12944-022-01734-7)
Supplement: Supplementary file 1 — Additional file 1: Figure S1. Detection of nonlinear relationships between BMI and lymph node metastasis. a Using the restricted cubic spine (RCS) function in the logistic regression model did not observe a nonlinear association between the BMI and lymph node metastasis risk. b RCS analysis with adjustment of sex, age, tumor location, grade, T stage, PNI, LVI, ELN, tumor size, and Lauren type. FigureS2. Bioinformatic analysis of the GSE84437 cohort. a Identification of DEGs between N0 and N+ patients in the GSE84437 cohort. b GO enrichment analysis of DEGs that were derived from the GSE84437 cohort. [file 12944_2022_1734_MOESM1_ESM.doc]

**Title:** Obesity promotes lipid accumulation in lymph node metastasis of gastric cancer: A retrospective case‒control study

**Author information**

Jian Xiao ^1^, Kuan Shen ^1^, Kanghui Liu ^1^, Yuanhang Wang ^1^, Hao Fan ^1^, Xinyi Zhou ^1^, Quan Chen ^1^, Li Hu ^1^, Gang Wang ^1^, Zekuan Xu ^1^, Li Yang ^1, 2^

^1^ Department of General Surgery, The First Affiliated Hospital of Nanjing Medical University, Nanjing, Jiangsu Province, China.

^2^ Department of General Surgery, Liyang People’s Hospital, Liyang Branch Hospital of Jiangsu Province Hospital, Liyang, Jiangsu Province, China.

**Corresponding author:** *Li Yang* E-mail: [pwkyangli@njmu.edu.cn](mailto:pwkyangli@njmu.edu.cn)

**Supplementary Figures**

**
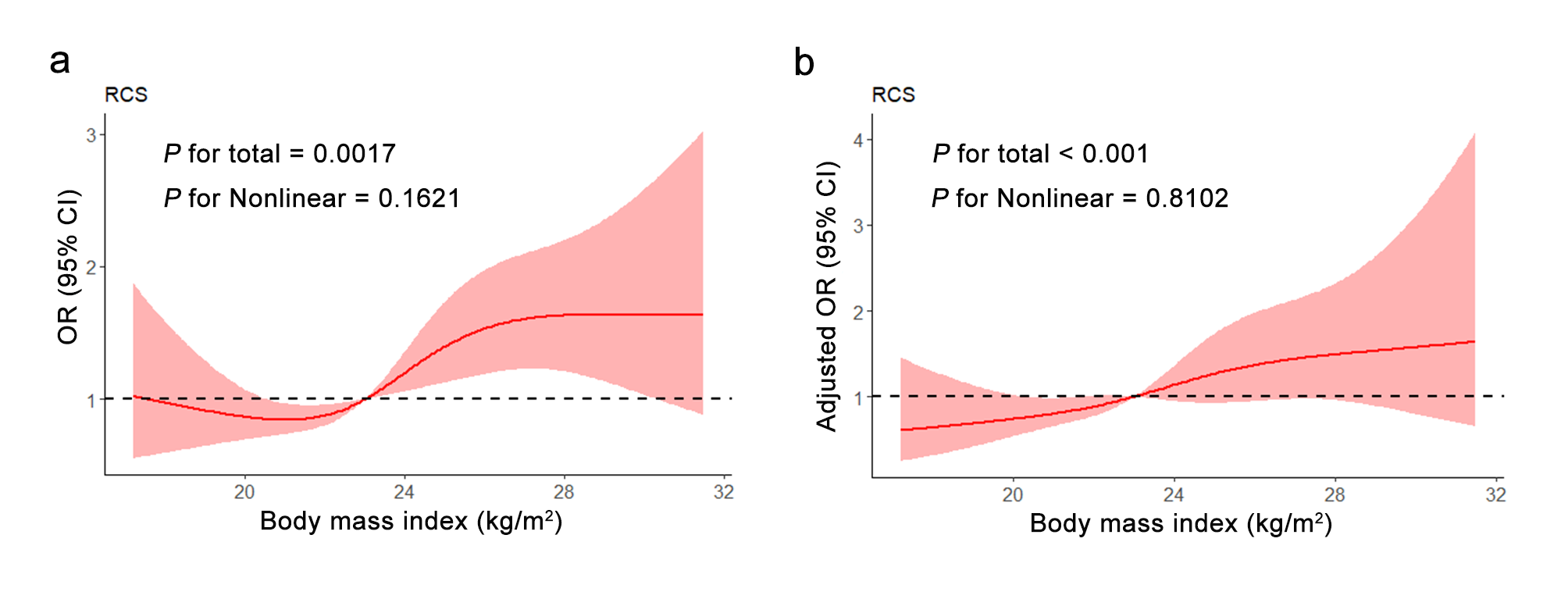
**

**Figure S1** Detection of nonlinear relationships between BMI and lymph node metastasis

**a** Using the restricted cubic spine (RCS) function in the logistic regression model did not observe a nonlinear association between the BMI and lymph node metastasis risk. **b** RCS analysis with adjustment of sex, age, tumor location, grade, T stage, PNI, LVI, ELN, tumor size, and Lauren type.


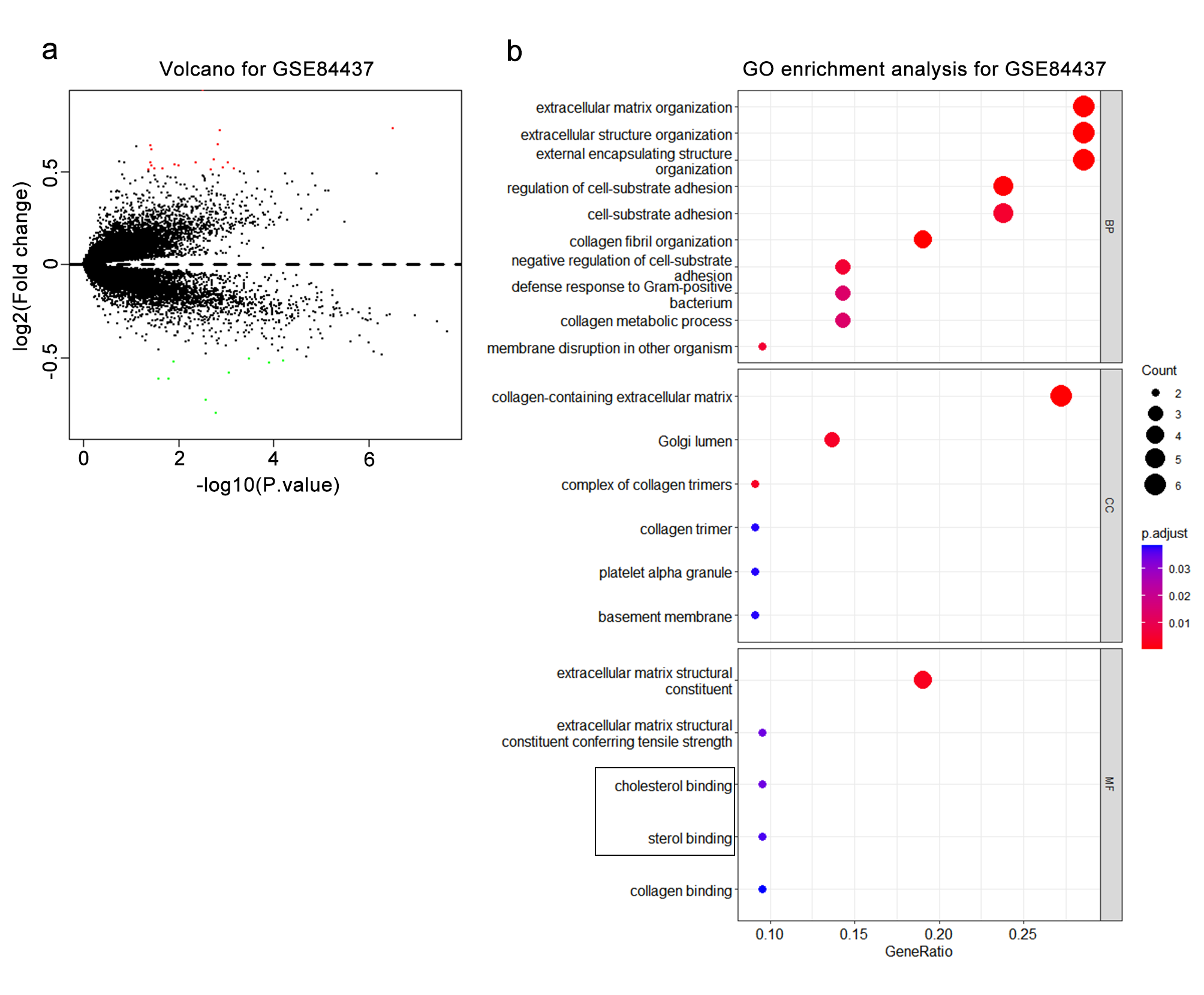


**Figure S2** Bioinformatic analysis of the GSE84437 cohort

**a** Identification of DEGs between N0 and N+ patients in the GSE84437 cohort. **b** GO enrichment analysis of DEGs that were derived from the GSE84437 cohort.
