# Supplementary material for: Obesity promotes lipid accumulation in lymph node metastasis of gastric cancer: a retrospective case‒control study
Source: Lipids Health Dis. 2022 Nov 17;21:123. doi: 10.1186/s12944-022-01734-7 (PMC9673345; doi:10.1186/s12944-022-01734-7)
Supplement: Supplementary file 2 — Additional file 2: Supplementary Table 1. Subgroup analysis for the association between categorized BMI and lymph node metastasis. [file 12944_2022_1734_MOESM2_ESM.docx]

**Supplementary table 1** Subgroup analysis for the association between categorized BMI and lymph node metastasis.

| Subgroups | 23-24.9 vs. < 23 (kg/m^2^) | |  | ≥ 25 vs. < 23 (kg/m^2^) | | *P* for interaction |
| --- | --- | --- | --- | --- | --- | --- |
|  | Adjusted OR (95% CI) ^†^ | *P* value ^†^ |  | Adjusted OR (95% CI) ^†^ | *P* value ^†^ |  |
| Sex |  |  |  |  |  | 0.774 |
| Male | 2.10 (1.15-3.84) | **0.015** |  | 2.05 (1.15-3.64) | **0.014** |  |
| Female | 1.88 (0.75-4.70) | 0.179 |  | 1.46 (0.54-3.96) | 0.453 |  |
| Age (years) |  |  |  |  |  | 0.551 |
| ≤ 65 | 1.93 (1.00-3.74) | 0.050 |  | 1.86 (0.99-3.52) | 0.056 |  |
| > 65 | 2.68 (1.23-5.83) | **0.013** |  | 1.87 (0.83-4.26) | 0.134 |  |
| Grade |  |  |  |  |  | 0.317 |
| G2 | 1.16 (0.37-3.65) | 0.795 |  | 0.86 (0.25-2.93) | 0.812 |  |
| G2-3 | 3.32 (1.33-8.28) | **0.010** |  | 4.65 (1.91-11.32) | **0.001** |  |
| G3 | 1.88 (0.87-4.08) | 0.111 |  | 1.35 (0.60-3.03) | 0.473 |  |
| Location |  |  |  |  |  | 0.489 |
| Upper | 4.06 (1.11-14.91) | **0.035** |  | 2.06 (0.59-7.22) | 0.258 |  |
| Middle | 1.39 (0.61-3.15) | 0.432 |  | 1.64 (0.67-4.07) | 0.279 |  |
| Lower | 2.20 (0.99-4.88) | 0.054 |  | 2.25 (1.03-4.91) | **0.041** |  |
| T stage |  |  |  |  |  | 0.951 |
| T1+T2 | 1.82 (0.97-3.43) | 0.061 |  | 2.05 (1.07-3.92) | **0.031** |  |
| T3+T4 | 2.14 (0.97-4.72) | 0.060 |  | 1.88 (0.90-3.93) | 0.094 |  |

*OR* odds ratio, *CI* confidence interval. *LDL-C* low-density lipoprotein cholesterol, *LP (a)* lipoprotein (a).

^†^ Adjusted for TG, TC, HDL-C, LDL-C, LP (a), THR, BMI, age, sex, location, grade, T stage, tumor size, PNI, LVI, Lauren classification, and ELN (excluding the stratified factor in each stratum).
